# Nutrients and Diet: A Relationship between Oxidative Stress, Aging, Obesity, and Related Noncommunicable Diseases

**DOI:** 10.1155/2018/7460453

**Published:** 2018-07-16

**Authors:** Rodrigo Valenzuela, Undurti N. Das, Luis A. Videla, Carolina G. Llorente

**Affiliations:** ^1^University of Chile, Santiago, Chile; ^2^BioScience Research Centre, GVP College of Engineering Campus and GVP Hospital and Medical College, Visakhapatnam, India; ^3^University of Granada, Granada, Spain

Nowadays, the significant increase in the prevalence of obesity worldwide is directly associated with an increased risk of developing noncommunicable diseases such as diabetes, cardiovascular disease, dyslipidemias, inflammatory disease, cancer, and even neurodegenerative diseases. In this regard, obesity and chronic diseases are not only limited to developing countries, nor to the adult population. On the contrary, the greatest increase in the incidence of obesity in the last 20 years has been observed in developing countries and young population.

Important risk factors involved in obesity development comprehend (i) high-energy intake; (ii) excessive fat consumption (especially saturated fat and transfatty acids); (iii) excessive intake of simple carbohydrates (sucrose, glucose, and fructose); (iv) insufficient intake of vegetables and fruits (sources of natural antioxidant), legumes (dietary fiber), and fish and seafood (sources of polyunsaturated fatty acid); and (v) unhealthy lifestyle (sedentarism among other factors like consumption of tobacco, alcohol, and drugs). These factors directly favor oxidative stress, which is a relevant metabolic disturbance related to the development of other pathologies.

In this sense, diet chemical composition and food preparation play a relevant and direct role in regulating several of the metabolic and molecular pathways involved in the prevention and treatment of obesity and its related comorbidities. For this reason, it is necessary to identify and understand molecular pathways involved in these events in order to develop nutritional strategies that contribute to the prevention and treatment of oxidative stress linked with the obesity.

In this special issue, the impact of specific diet and nutritional interventions destined to prevent or attenuate obesity-related oxidative stress injuries is presented. By reading the different articles, readers will be able to identify relevant aspects underlying molecular changes generated by oxidative stress as a consequence of obesity in different study models. The risk of developing metabolic syndrome and cardiovascular alterations in humans, as well as dietary interventions using foods or bioactive compounds (polyphenols or other micronutrients) to treat these alterations, is addressed in this issue. It should be noted that some of the studies published here suggest the protective effect of bioactive compounds and exercise on oxidative stress and other alterations generated by obesity. Thus, in this special issue, results about reproduction, oxidative stress, aging, obesity, and related noncommunicable diseases and their interaction with nutrients and diet are reported.

*Rodrigo Valenzuela* *Undurti N. Das* *Luis A. Videla* *Carolina G. Llorente*

